# Ta-YOLO: overcoming target blocked challenges in greenhouse tomato detection and counting

**DOI:** 10.3389/fpls.2025.1618214

**Published:** 2025-07-08

**Authors:** Yun Zhao, Yijia Chen, Xing Xu, Yong He, Hao Gan, Na Wu, Zhechen Wang, Xi Sun, Yali Wang, Petr Skobelev, Yanan Mi

**Affiliations:** ^1^ School of Artificial Intelligence and Information Engineering, Zhejiang University of Science and Technology, Hangzhou, China; ^2^ College of Biosystems Engineering and Food Science, Zhejiang University, Hangzhou, China; ^3^ Department of Biosystems Engineering and Soil Science, University of Tennessee, Knoxville, TN, United States; ^4^ Cardiovascular Medicine, Zhejiang Hospital, Hangzhou, China; ^5^ Samara Federal Research Scientific Center, Russian Academy of Sciences, Samara, Russia; ^6^ Department of Business Development, Pegasor Oy, Tampere, Finland

**Keywords:** machine vision, Ta-YOLO, target detection, tomato counting, target blocked

## Abstract

Screening and cultivating healthy small tomatoes, along with accurately predicting their yields, are crucial for sustaining the economy of tomato industry. However, in field scenarios, counting small tomato fruits is often hindered by environmental factors such as leaf shading. To address this challenge, this study proposed the Ta-YOLO modeling framework, aimed at improving the efficiency and accuracy of small tomato fruit detection. We captured images of small tomatoes at various stages of ripeness in real-world settings and compiled them into datasets for training and testing the model. First, we utilized the Space-to-Depth module to efficiently leverage the implicit features of the images while ensuring a lightweight operation of the backbone network. Next, we developed a novel pyramid pooling module(DASPPF) to capture global information through average pooling, effectively reducing the impact of edge and background noise on detection. We also introduced an additional tiny target detection head alongside the original detection head, enabling multi-scale detection of small tomatoes. To further enhance the model’s focus on relevant information and improve its ability to recognize small targets, we designed a multi-dimensional attention structure(CSAM) that generated feature maps with more valuable information. Finally, we proposed the EWDIoU bounding box loss function, which leveraged a 2D Gaussian distribution to enhance the model’s accuracy and robustness. The experimental results showed that the number of parameters, FLOPs, and FPS of our designed Ta-YOLO were 10.58M, 14.4G, and 131.58, respectively, and its mean average precision(mAP) reached 84.4%. It can better realize the counting of tomatoes with different maturity levels, which helps to improve the efficiency of the small tomato production and planting process.

## 1 Introduction

Small tomatoes are a flavorful, nutritious crop with high economic value and important in the global vegetable trade. China’s small tomato industry has grown rapidly over the past 20 years, with more than 30,000 acres planted nationwide, jumping to the top spot in the world (Guan et al., 2018). The huge economic benefits have made it economically important to accurately estimate the number of fruits before harvest. On one hand early yield estimation can help producers adjust their planting strategies. On the other hand, it can also effectively improve the operators’ income and operation development strategies. However, estimating the number of small tomatoes is greatly challenged by their own tight growth, dense leaf shade, and short ripening period. Traditional manual methods of counting are not only economically costly, but also time-consuming and easily hindered by human error and subjectivity. These problems can easily compromise the accuracy of the counting of data. Therefore, it is very important to utilize robotics to achieve an automated and scalable approach to improve the accuracy and speed of fruit detection and counting in agriculture (Zhao et al., 2022).

In recent years, with the development of deep learning, computer vision technology is highly integrated with the agricultural industry. In the field of computer vision, it mainly includes a variety of tasks, such as image classification, target detection, entity segmentation, etc. Among them, target detection is able to locate the target in the form of a rectangular box, which has high accuracy and real-time performance (Srinivas et al., 2016). Therefore, target detection technology is most widely used in agricultural fruit detection and counting, and also provides a new solution for the application of robots in agriculture.

Deep learning based target detection algorithms include single-stage and two-stage algorithms. The single-stage algorithms realize the detection process through a single network branch, eliminating the complex steps such as feature extraction and generation of candidate frames in the two-stage. Therefore, single-stage target detection algorithms are famous for their fast detection. Currently, excellent single-stage target detection algorithms include SSD (Liu et al., 2016), RetinaNet (Lin et al., 2017), YOLOv5 (Redmon et al., 2016), YOLOv8, YOLOv9 (Wang et al., 2024), and the latest YOLOv11 (Khanam and Muhammad, 2024). Two-stage detection algorithms first generate a large number of candidate regions containing the target object, and then perform further processing such as region classification, bounding box regression, and so on for each candidate region. Classical two-stage detection algorithms include R-CNN (Girshick et al., 2014), Fast R-CNN (Girshick, 2015), Faster R-CNN (Ren et al., 2016), Mask R-CNN (He et al., 2017), Cascade R-CNN (Cai and Vasconcelos, 2018), and DetectoRS (Qiao et al., 2021). However, when facing complex scenes, although two-stage target detection algorithms are able to provide higher accuracy, it has a large computational overhead, which makes it unsuitable for a wide range of scenarios such as real-time detection.

Therefore, researchers must balance the advantages and disadvantages of the two algorithms in light of practical needs, selecting and enhancing them accordingly. These algorithms have been widely used for the recognition of a variety of crops, such as potato (Johnson et al., 2021), maize (Khaki et al., 2020), rice (Zhang et al., 2022), apple (Wang and He., 2022), and so on. For the detection of small tomato crop, Seo et al. (Seo et al., 2021) proposed a real-time robotic detection system based on Faster R-CNN for detecting tomato growth and selecting a color model that is robust to external light to develop an image-based ripeness criterion for tomato fruits. Wang et al. (Wang et al., 2022) designed an improved Faster R-CNN model, MatDet, for tomato ripeness detection to address the difficulty of detecting tomato ripeness in complex scenes by using RolAlign to obtain more accurate bounding boxes in the feature mapping stage. Wang et al. (Wang et al., 2023) proposed an R-CNN model for tomato detection and segmentation tasks, using Swin Transformer as the backbone network for better feature extraction, the method can not only effectively recognize tomato in cherry tomato varieties, but also differentiate between different ripening stages. The introduction of the YOLO (You Only Look Once) family of models provides the advantage of directly predicting the entire image without generating candidate regions and has also been widely used by researchers. Lawal et al. (Lawal, 2021) used an improved YOLOv3 model to realize the detection of tomato counts in natural scenes, and solved the problem of gradient vanishing during model training by introducing the MixNet backbone network. Miao et al. (Miao et al., 2023) proposed an algorithm for estimating the ripeness of individual tomato clusters and an integrated method for locating tomato stems based on experimental errors using the YOLOv5 network architecture. Liu et al. (Liu et al., 2020) proposed a tomato detection model called “YOLO-tomato” using the improved YOLOv3 architecture, which utilizes a circular bounding box instead of the traditional rectangular bounding box for tomato localization, which reduces the predicted coordinates and thus achieves more accurate tomato matching. In (Ge et al., 2022), a detection model named “YOLO-deepsort” is proposed to realize the periodic detection of tomato growth, and the effective features are enhanced by using BiFPN multiscale fusion structure to realize the improvement of detection accuracy. In addition, the combination of robots and inspection algorithms brings a number of significant advantages to the field of tomato inspection. Dai et al. (Dai et al., 2022) proposed a tomato fruit counting algorithm for greenhouse inspection robots, which tracks the position of tomatoes in the image by the spatial displacement information of the robot, while 3D depth filtering is used to avoid the interference of complex backgrounds on tomato counting. Rong et al. (Rong et al., 2023) Proposed an improved tomato cluster counting method based on YOLOv4, which incorporates target detection, multi-target tracking, and region-specific tracking counting in a robot to reduce the problem of tracked tomato cluster offset. Li et al. (Li et al., 2023) based on the improved YOLOv8 model, the MHSA attention mechanism is utilized to enhance the ability of the network to extract diverse features, and at the same time, it is mounted on the robot to realize the real-time hierarchical detection and counting function in the real scene, and achieve good detection results. Ruparelia et al. (Ruparelia et al., 2022) proposed a deep learning based tomato detection system for distinguishing between healthy, ripe and unripe tomatoes using different versions of the YOLO architecture.

However, practical applications of tomato detection and counting still face significant challenges under occlusion conditions. Fruits obstructed by other fruits, leaves, calyxes, stems, and similar structures can substantially degrade the accuracy of vision-based robotic detection systems. Specifically, the following issues are observed: (1) During the late fruit-setting stage, the extremely small size of tomato fruits increases the risk of missed detections; (2) In the fruiting stage, the dense distribution of small tomatoes combined with extensive occlusion frequently results in undetected instances; (3) Occlusion by branches, leaves, and stems can lead to false positives during the fruiting stage; (4) Leaf shading during fruiting may also cause both false detections and omissions of small tomatoes. To address these challenges, this study explores the integration of feature representations at varying depths across different branching structures to enhance the detection of small tomato targets through the fusion of multi-level feature information.

In summary, this study proposed a small tomato target detection method based on the YOLOv8 network architecture, specifically designed to address the occlusion challenges encountered during the counting of small tomato fruits in large-scale production environments. The main contributions of this research are outlined as follows:

1. Images of small tomatoes at different maturity stages were collected under large-scale cultivation conditions to construct a real-world small tomato dataset. The tomatoes in each image were annotated and categorized into three distinct maturity levels. To improve the robustness and generalization of the detection model, the dataset was further augmented using a set of simple yet effective data augmentation techniques applied to both the images and their corresponding annotations.
2. In real-world scenarios, the growth of small tomatoes is often accompanied by dense foliage and branching, leading to challenges such as the loss of fine-grained image features during recognition. To address these issues, this study incorporated a C2f-RepGhost module combined with a Space-to-Depth convolutional structure, enabling the proposed Ta-YOLO model to preserve detailed feature representations while maintaining a lightweight design. Furthermore, an additional detection head was introduced to enhance the model’s capacity for small object feature extraction. To further mitigate the impact of peripheral edge information on core feature representation, a Dilated Atrous Spatial Pyramid Pooling Fusion (DASPPF) module was integrated into the architecture.
3. This study proposed a CSAM attention mechanism, which integrates spatial and channel attention to enhance the model’s focus on salient features. By jointly leveraging spatial and channel-wise dependencies, the CSAM module improves the model’s sensitivity to occluded regions and enhances its capability to accurately recognize targets under complex occlusion conditions.
4. Traditional IoU-based loss functions often exhibit substantial bias when handling objects of varying scales. To mitigate this issue, we proposed the EWDIoU bounding box regression loss, which models the distance between the predicted box and the ground truth using a two-dimensional Gaussian distribution. This formulation enhances the model’s sensitivity to small target regions, thereby reducing scale-related bias and improving both the recognition accuracy and overall robustness of the detection framework.
5. The proposed model demonstrates effective detection and counting of small tomatoes in real greenhouse environments. It successfully addressed the occlusion challenges associated with short growth periods and validates the efficacy of the Ta-YOLO architecture in practical agricultural scenarios for accurate and robust small tomato detection and counting.

## 2 Materials and methods

### 2.1 Dataset acquisition and processing

**Figure 1:**
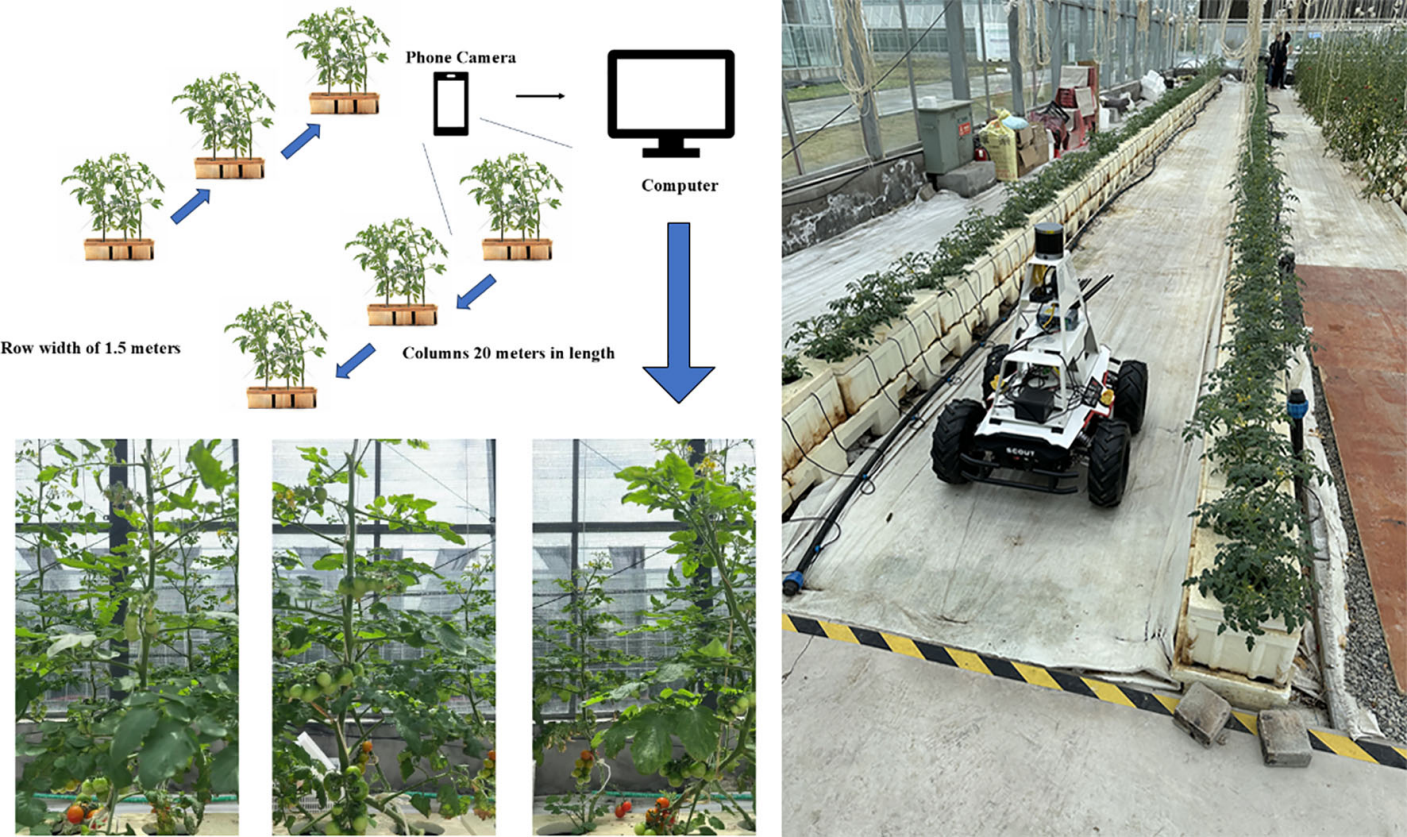
Schematic of little tomato dataset acquisition and dataset collection tools.

Existing tomato datasets primarily consist of images featuring single or multiple tomatoes against relatively clean and unobstructed backgrounds, limiting their applicability to real-world field production scenarios. Therefore, this study collected data from a small tomato cultivation base located at the International Internet Agricultural Expo Park in Wuzhen City, Zhejiang Province, where tomatoes at various growth stages were cultivated for market supply. Data acquisition was conducted over the period from April 3 to May 30, 2024. A SCOUT 2.0 robot equipped with an iPhone 14 Pro mounted horizontally was utilized to capture images of the small tomato plants. A total of 160 plants were arranged in two rows, each extending 20 meters in length. During data collection, the robot moved at a constant speed, photographing each row sequentially from left to right and then returning from right to left to capture images in the opposite direction. The acquired images were subsequently uploaded to a PC for further processing. The overall experimental setup is illustrated in **Figure 1**, with the right panel depicting the robot in operation.

**Figure 2:**
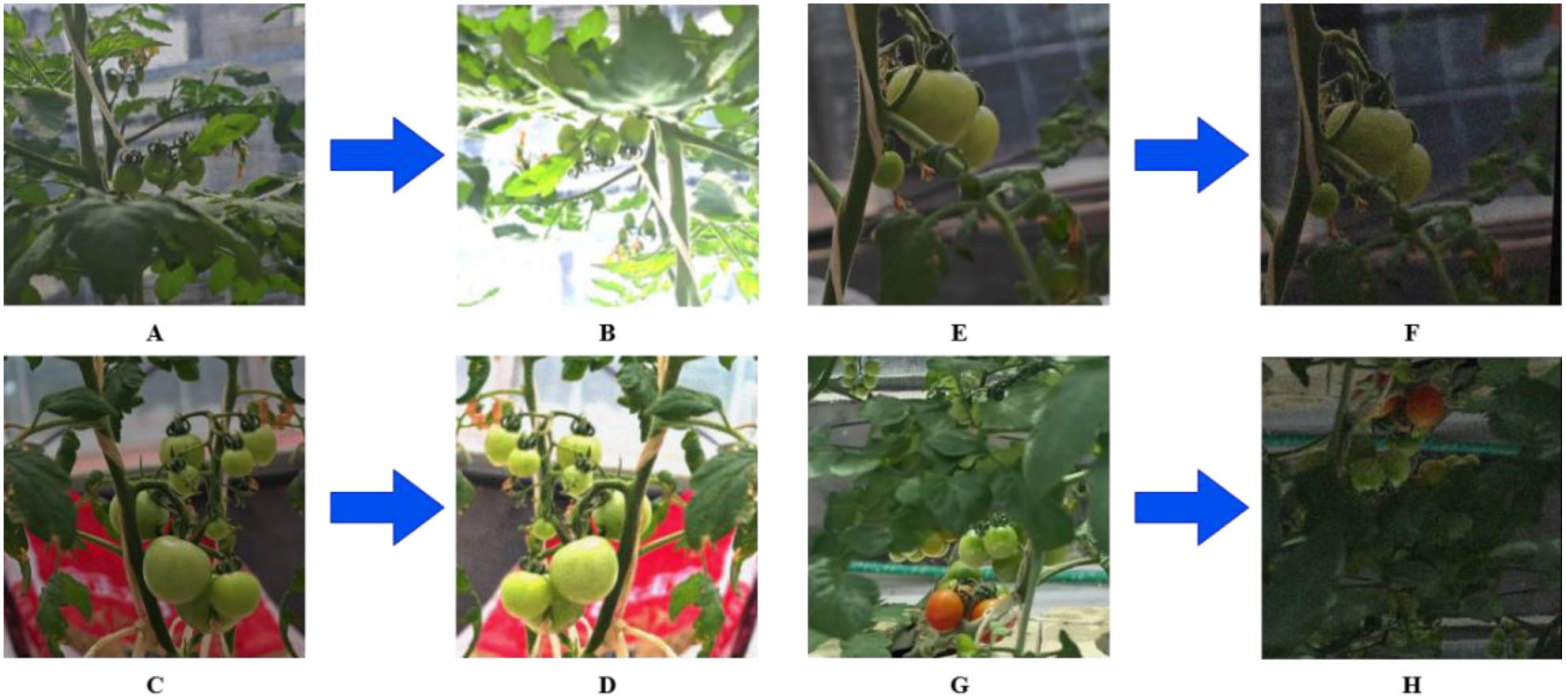
Example of 4 forms of blocked tomatoes images. **(A)** Example of extreme tiny tomatoes image. **(B)** Enhanced extreme tiny tomatoes image. **(C)** Example of mutual shading between classes image. **(D)** Enhanced mutual shading between classes image. **(E)** Example of branch stalk shading image. **(F)** Enhanced branch stalk shading image. **(G)** Example of leaf shading image. **(H)** Enhanced leaf shading image.

The photographed images were standardized to 640*640 pixels, labeled using the LabelImg tool, with the following labels: green fruit tomato, red fruit tomato and yellow fruit tomato. Following agronomic standards and harvesting requirements, these three labels correspond to unripe tomatoes, ripe tomatoes, and tomatoes between unripe and ripe stages, respectively. Such three classifications can fit the actual production decisions and reduce redundant judgements, while ensuring the efficiency of data annotation and data processing (Wan et al., 2018). Finally, the original small tomato data samples were obtained as 661, which were divided into training set, validation set and test set according to the ratio of 3:1:1. Given that the training set consists of only 535 images with only a small portion of overexposure and blurring under natural light conditions, in order to enhance the generalization of the model, six data enhancement techniques were adopted to process the training set data, including exposure, rotation, blurring, random brightness adjustment, mirroring and noise addition. As shown in **Figure 2**, each image is enhanced by taking a random combination of three of the above enhancement methods.

A notable feature of this dataset is the inclusion of complex distractions from real environments, with varying degrees of occlusion problems on each image. Based on the type of blocked, we grouped the detection difficulties into four categories: extreme tiny tomatoes, mutual shading between classes, branch stalk shading, and leaf shading. The proportion of tiny small tomatoes was the largest, with a more similar amount of interclass shading and leaf shading, and a relatively small amount of branch and stem shading.(The number of different maturity categories in the original dataset and the corresponding number for each shading type are shown in **Table 1**) In order to mitigate the impact that category imbalance would have on training, we used the CopyPaste method to perform an additional data augmentation operation on red and yellow fruit tomatoes, which is to copy the instances in the image containing red and yellow fruit tomatoes and paste them into another image during the training process, adding instances from fewer categories to generate new training samples.

**Table 1:** Number of different maturity categories and the corresponding number for each type of shading.

Categories	Instances	Tiny	Classes shading	Stalk shading	Leaf shading
gtomato	9973	2069	885	455	809
rtomato	1853				
ytomato	1220				

### 2.2 Hardware design

**Figure 3:**
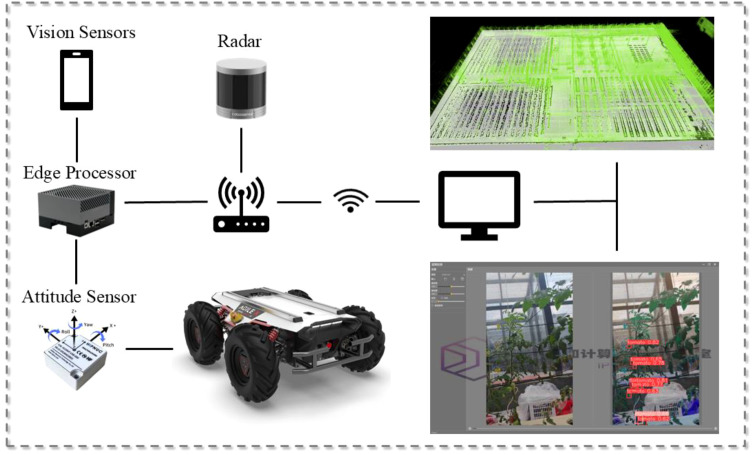
Compositional architecture of the robot as well as map construction of the whole scene and real-time detection results in real applications.

Combined with the growth characteristics of the small tomato itself, during the fruiting period, the growth height of the small tomato ranges from 0.5 to 2.3 meters, and within a relatively short period of time, there is a large span of height change. In order to be able to meet the normal work at different heights, we have also designed and improved the agricultural robot hardware accordingly. Firstly, the robot stand is built by 1.5mm iron plate, and the overall structure is in the shape of a tower, which is divided into three layers to meet the needs of different sensors and different heights of mounting. Next, electrical adapter devices are fixed on the bottom layer for powering the sensors of each device and edge computing devices are fixed on the bottom layer for processing real-time data. To improve the stability of the collected data, the camera head is mounted on the bottom tail, and the shooting camera is mounted on the camera head tilt rotation connector. The middle layer installs the router used by the robot for communication, which facilitates remote operation and control of the robot. The top layer is fitted with LIDAR to prevent other equipment from interfering with the laser. In this work, we deployed the detection algorithm ultimately on an edge device and utilized an agricultural robot to achieve the work of detecting and counting small tomatoes of different ripeness in a facility greenhouse, overcoming the problem of occlusion during the growing process. The detailed hardware composition as well as the field applications are shown in **Figure 3**.

### 2.3 Small tomatoes detection based on Ta-YOLO

In real production environments, the large-scale planting of small tomatoes has significant advantages in improving production efficiency, but in the growth process of small tomatoes, regularized planting makes the lush branches and leaves obscure the fruits, and changes in the intensity and angle of the sunlight at different moments also significantly change the brightness and contrast of the image, making it more difficult to count the fruits.

**Figure 4:**
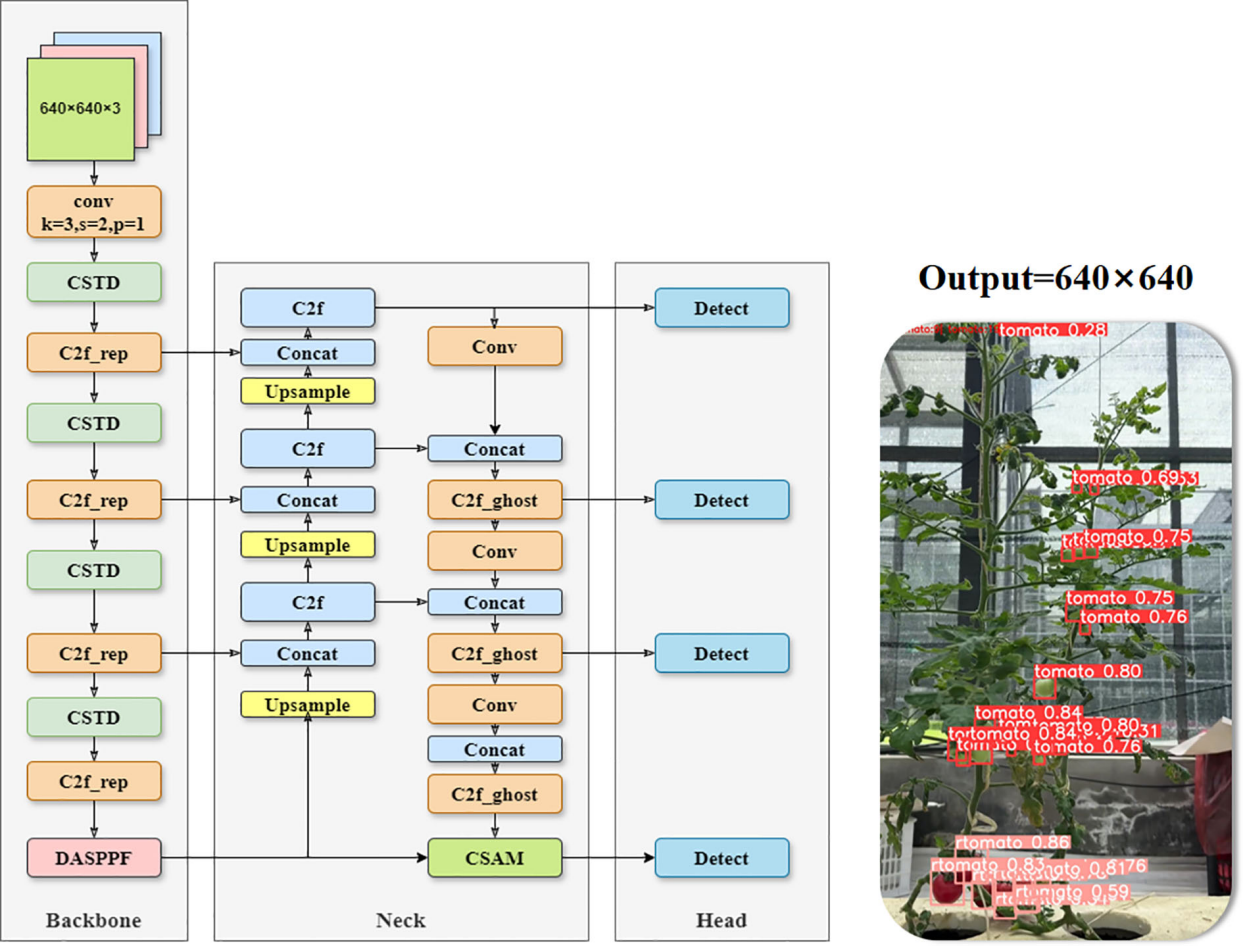
Overall structure of Ta-YOLO model.

This study proposed a Ta-YOLO model for the detection and counting of small tomatoes in a real production environment to address these challenges. The model retained the overall framework of YOLOv8n, adopted C2f-rghost combined with Space-to-Depth Conv module to reconstruct the backbone structure, and at the same time, the DASPPF structure was proposed to enhance the fine-grained representation. And the CSAM multiple attention mechanism was created in the neck structure, and an additional detection head was added to enhance the detection ability in different scales and occlusion situations. Finally, the EWDIoU loss function was proposed to improve the detection accuracy for small tomatoes. The overall structure of Ta-YOLO is shown in **Figure 4** These improvements will be further illustrated above.

#### 2.3.1 Lightweight network design

**Figure 5:**
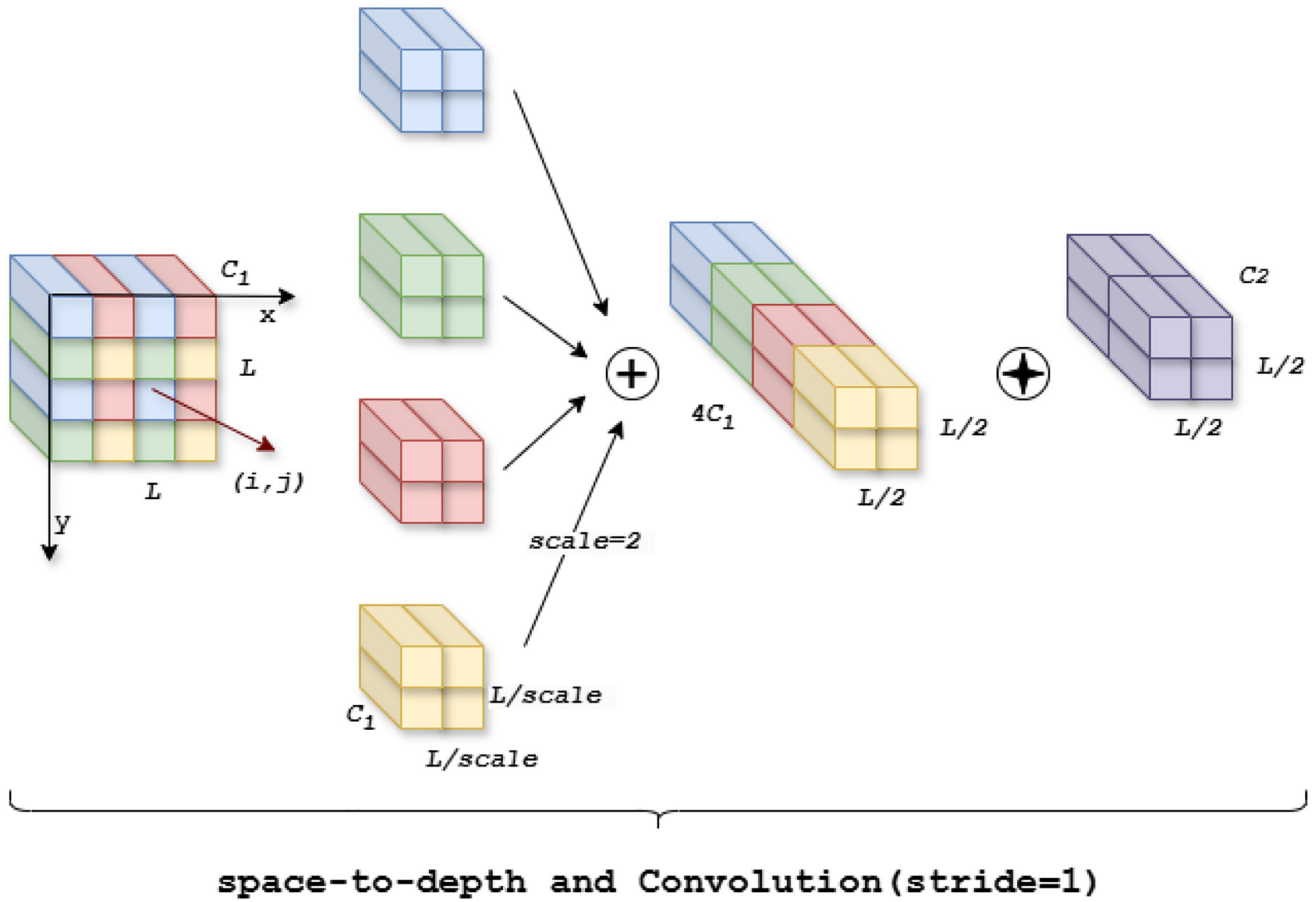
Schematic of SPDC module when scale = 2.

In the YOLOv8 backbone network, Convolutional Neural Networks (CNNs) perform well in different tasks such as classification and detection. However, due to the use of pooling layers, connecting across steps, and other operations in the CNN architecture, which allows the model to easily skip over a large amount of redundant pixel information, it is not possible to learn a more efficient representation of the features. Therefore, in our model we use the Space-to-Depth (Sunkara and Luo, 2022) and Conv module, which consists of a space-to-depth (SPD) layer and a convolution-free step (Conv) layer (shown in **Figure 5**). This method alters the image using downsampled feature maps within and across the CNN, allowing the model to reduce the sharp performance degradation when faced with small tomato targets.

For example, we denote the feature map with input size $\text{L}\times \text{L}\times C_{1}$ as X(i,j) , and the feature map can be divisible by all scales to get the feature subgraph x(i,j) . When scale=2 , we get four feature sub-feature maps, each of which has the shape of $\left(\frac{L}{2},\frac{L}{2},C_{1}\right)$ . Next, we splice these sub-feature maps along the channel dimensions to get a new feature map $X^{\prime }\left(\frac{L}{scale},\frac{L}{scale},\;scale^{2}C_{1}\right)$ , and a non-Stepwise convolution with a $C_{2}$ is added after the new feature map $\left(C_{2}<scale^{2}C_{1}\right)$ . Then, the new feature map is further transformed to get $X^{\prime \prime }\left(\frac{L}{scale},\frac{L}{scale},C_{2}\right)$ , which retains all the discriminative information as much as possible without reducing the feature map.

**Figure 6:**
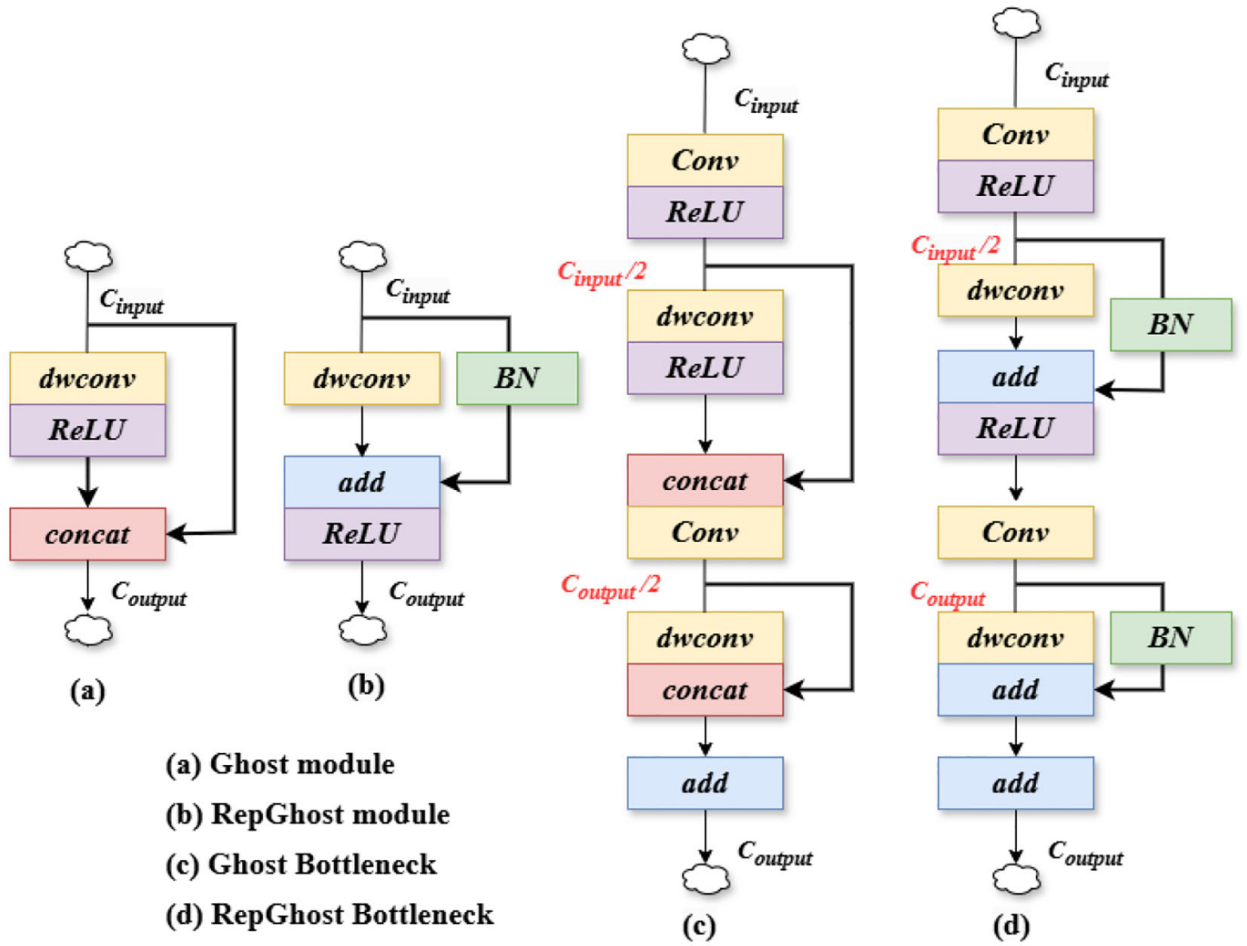
Schematic of lightweight backbone components. **(A)** Ghost module **(B)** RepGhost module **(C)** Ghost Bottleneck **(D)** RepGhost Bottleneck.

In target detection tasks, lightweight network structures tend to lower the computational cost and reduce the size of the model. In order to maintain the improved accuracy of small-target tomato detection without introducing additional computational parameters, we try to replace the traditional Bottleneck structure inside the C2f module with GhostBottleneck and Repghostbottleneck, which in turn, forms the C2f_Ghost module (Han et al., 2020) with the C2f_Repghost (Chen et al., 2022) module in **Figure 6**.

The C2f_Ghost module expands the number of channels by utilizing the underlying residual structure, while reducing the number of channels that need to be shortcut connected. This design not only optimizes the network structure, allowing for a reduction in the amount of computation, but also preserves the necessary feature representation to improve efficiency without losing the more obvious accuracy. Unlike the C2f_Ghost module, the C2f_Repghost module reduces the number of intermediate channels and downsamples the feature maps with a reduced number of channels. This further improves the computational efficiency and makes the model more efficient. At this point, the feature maps also capture the long-distance dependence between pixels in different spatial locations, which enhances the expressive power of the model (Wu et al., 2024). Especially in resource-constrained environments, this lightweight structural design, by generating a large number of lightweight feature maps, not only enables efficient dissemination of information, but also provides rich feature representations for the subsequent layers. Also, it avoids the computational bottleneck in traditional convolution and, reduces the computational overhead. Thus it saves memory and, ensures that the model reduces the resource consumption of hardware while maintaining high performance. Additionally, it, and also lays a good foundation for subsequent model deployment and migration.

#### 2.3.2 Enhanced feature fusion for CSAM multiple attention structures

In the Neck of YOLOv8, multi-scale feature fusion is usually performed using a feature pyramid network (Zhao et al., 2023). However, feature map fusion in this part often relies on relatively small convolutional operations, resulting in a limited sense field. As the depth of the network increases, the desire to acquire a larger range of features leads to a decrease in the learning rate of the model and the transfer of feature information becomes difficult. In order to better fuse meaningful features in the channel and spatial dimensions and increase the network information effectiveness, we propose an innovative CSAM multi-attention structure that combines Non-Local positional attention (Wang et al., 2018) with the channel attention mechanism to achieve deep aggregation of spatial information in feature mapping. In the CSAM structure, we first halve the number of channels of the input feature map, which not only helps to reduce the computational redundancy and the subsequent computational burden, but also effectively promotes the selective focusing of features, making the subsequent attention mechanism more targeted and efficient. Subsequently, we apply positional attention and channel attention operations on the feature maps that have been halved by the number of channels, and use average pooling and maximum pooling operations to gather effective information, which is subsequently shared into the MLP to effectively integrate the captured important features, enabling the structure to adaptively weight the features according to the contextual information and expand the sensory field (Zhao et al., 2012). In particular, it can better enhance the global information when facing the lack of local feature information for small target tomatoes. Then the number of channels of the processed feature map is restored to the original size, preserving the network’s ability to capture high-dimensional features.


(1) $$\begin{array}{c} F_{\left(a,b\right)}=\gamma \left(F_{in}\right) \end{array}$$

(2) $$\begin{array}{c} CA\left(F_{a}\right)=\sigma \left(MLP\left(\varphi_{1}\left(F_{a}\right)\right)+MLP\left(\varphi_{2}\left(F_{a}\right)\right)\right) \end{array}$$

(3) $$\begin{array}{c} F_{a1}=CA\left(F_{a}\right)⊕F_{a} \end{array}$$

Equations 1–10 represents the CSAM calculation process. Where γ denotes a split operation that halves the number of channels, $F_{\alpha }$ denotes the feature map at each stage, CA denotes the imposition of a channel attention mechanism, SA denotes the imposition of a spatial attention mechanism, and σ denotes a sigmoid operation, $\varphi_{1}$ denotes the maximum pooling operation, $\varphi_{2}$ denotes the average pooling operation, and ⊕ denotes the feature map summation operation.

**Figure 7:**
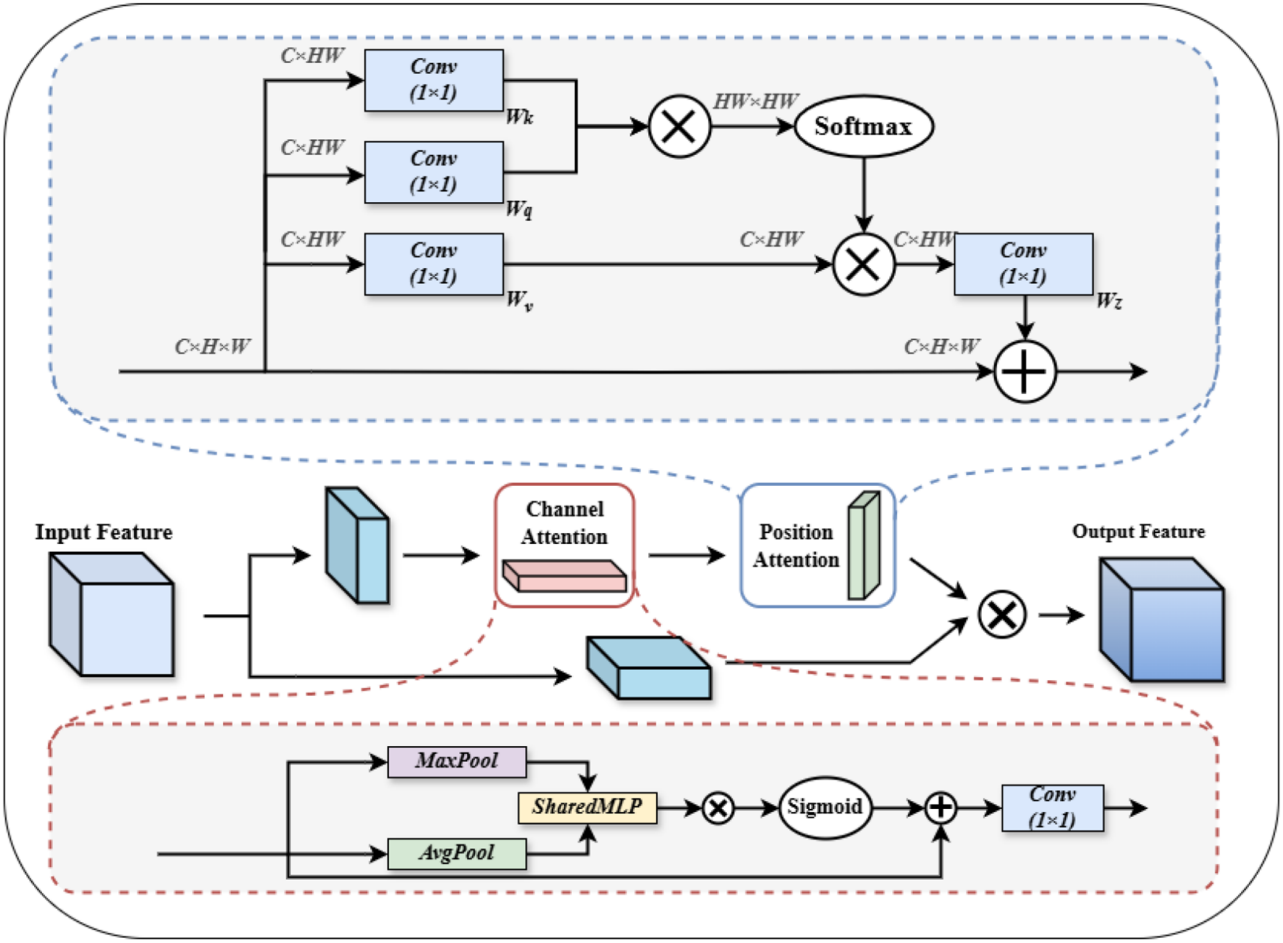
CSAM overall structure.


In order to realize the dependence of different positional information of feature map on other positional information in the surrounding area, and to expand the range of features obtained by ourselves, we carry out Reshap operation on the feature map $F_{a1}\in R^{C\times H\times W}$ outputted from channel attention to obtain $F_{a1}\in R^{C\times HW}$ for subsequent matrix operation. Then three linear mappings are performed separately using 1×1 convolution, i.e., $W_{v},W_{q},W_{k}$ in **Figure 7**.

(4) $$\begin{array}{c} f\left(x_{i},x_{j}\right)=h\left(x\right)Softmax\left[\theta {\left(x\right)}^{T}\omega \left(x\right)\right]\; \end{array}$$

(5) $$\begin{array}{c} \theta \left(x\right)=W_{q}x\; \end{array}$$

(6) $$\begin{array}{c} \omega \left(x\right)=W_{k}x \end{array}$$

(7) $$\begin{array}{c} g\left(x\right)=W_{v}x \end{array}$$

(8) $$\begin{array}{c} F_{i}=\frac{1}{C\left(x\right)}\sum_{\forall j}f\left(x_{i},x_{j}\right)g\left(x_{j}\right) \end{array}$$

(9) $$\begin{array}{c} F_{a2}=SA\left(F_{a1}\right)⊕F_{a1} \end{array}$$

(10) $$\begin{array}{c} F_{out}=g\left(F_{b}⊕F_{a2}\right) \end{array}$$

The corresponding linear transformations are denoted as h(x) , θ(x) , ω(x) , representing the modulation function, query projection, and key projection, respectively. After applying these transformations, the feature response of a given pixel to all other spatial positions is computed through a similarity-based attention mechanism, typically implemented via a softmax operation, followed by normalization and weighted summation. Specifically, θ(x) projects the input into a query representation, while ω(x) encodes key features to be compared against the query. The modulation function h(x) is optional and can be designed to incorporate spatial priors or learnable scaling factors. This mechanism enables each spatial location to adaptively aggregate contextual information from the entire feature map, thereby enhancing the network’s capacity to capture long-range dependencies. Here, $f\left(x_{i},x_{j}\right)$ denotes the affinity between position i and j , $g\left(x_{j}\right)$ extracts content features from position j , and C(x) serves as a normalization factor to ensure stability of the attention distribution.

To further strengthen representational capacity, we integrate spatial and channel attention mechanisms. The spatial attention emphasizes “where” to focus, enhancing the model’s sensitivity to informative regions even under partial occlusion. Meanwhile, the channel attention focuses on “what” to emphasize, selectively enhancing discriminative feature channels. The synergy of both attention types enables the model to infer occluded or ambiguous targets from contextual cues, significantly improving robustness and recognition accuracy in complex agricultural environments.

#### 2.3.3 EWDIoU loss functions

**Figure 8:**
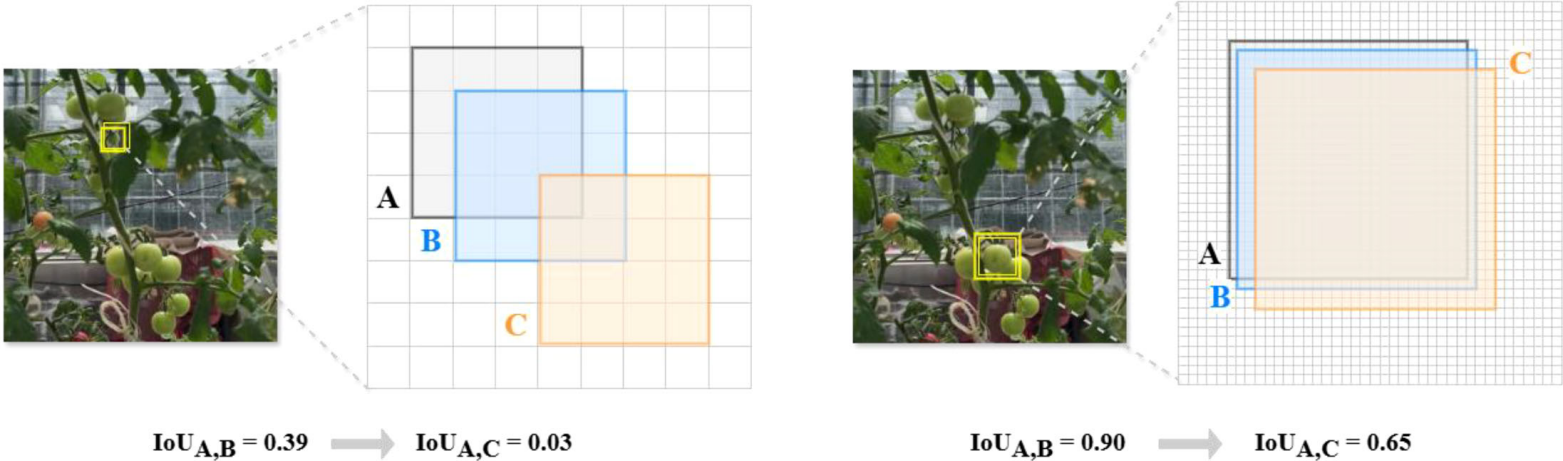
The figure shows the variation of target IoU for different pixel sizes. In the left figure **(A)** is the 4*4 pixel target real frame, **(B)** is the predicted frame with 1 pixel deviation, **(C)** is the predicted frame with 3 pixel deviation; In the right figure **(A)** is the real target of 45*45 pixels, **(B)** is the prediction frame with a deviation of 1 pixel and **(C)** is the prediction frame with a deviation of 3 pixels.

In target detection, IoU is often used to calculate the overlap ratio between the predicted frames and real frames. One issue this method has is that there is, a large difference in the sensitivity of IoU when applied to targets with different sizes. For example, for a small target object of 4×4 pixels, a small positional deviation leads to a significant decrease in IoU, whereas for a larger target object of 45×45 , the change in IoU is smaller for the same positional deviation, as show in **Figure 8**. This situation leads to insufficient learning of small target features by the model or stagnation of the training process, which does not allow the model to be fully optimized. This is because, the sensitivity of IoUs for objects of different sizes mainly stems from the particularity that the position of the enclosing box can only be changed in a discrete manner. To mitigate the situation where IoU can be significantly degraded in small-target tomato detection, we propose to use the EWDIoU loss function.

The original YOLOv8 uses CIoU for loss calculation which can only reflect the difference in the aspect ratio of the enclosing frame, not the width and height respectively. This may hinder the model to optimize effectively (Zhao et al., 2022). Due to this limitation in CIoU, the EIoU (Equation 11) added a penalty term to split the influence factor of the width and height ratios, and calculated the length and width of the target and predicted frames respectively (Zhao et al., 2023).


(11) $$\begin{array}{c} f_{eIoU}=1-IoU+\frac{\alpha^{2}\left(b,b^{gt}\right)}{{\left(d_{w}\right)}^{2}+{\left(d_{h}\right)}^{2}}+\frac{\alpha^{2}\left(w,w^{gt}\right)}{{\left(d_{w}\right)}^{2}}+\frac{\alpha^{2}\left(h,h^{gt}\right)}{{\left(d_{h}\right)}^{2}} \end{array}$$

Here b and $b^{gt}$ denote the centroids of the prediction frame and the real frame, respectively, $\alpha^{2}\left(\cdot \right)={\left\|b-b^{gt}\right\|}_{2}$ denotes the Euclidean distance between the two, and $d_{w}$ and $d_{h}$ denote the width and height of the smallest outer bounding box covering the two enclosing frames, respectively. However, the discrete nature of the change in the position of the enclosing box hinders the accuracy. So, we adopt a new metric to measure the similarity of the enclosing box by Wasserstein Distance (Wang et al., 2021) which replaces the partial EIoU. The hyperparameters are utilized to balance the coordination of the two IoU distances. Here, $\lambda_{1}+\lambda_{2}=1$ This way, it mitigates the high sensitivity of the normal IoU for small target tomatoes.


(12) $$\begin{array}{c} EWD_{IoU}=\lambda_{1}f_{eIoU}+\lambda_{2}\left(1-exp\left(-\frac{\sqrt{W_{2}^{2}\left(\mu_{a},\mu_{b}\right)}}{M}\right)\right) \end{array}$$

Firstly, we observe that in real planting scenarios, our annotation of the small tomato dataset tends to be in the form of a rectangular annotation box, where the body of the small tomato and the other background information will be distributed in the center and the edge of the bounding box. The importance of the pixel’s weight decreases from the center to the edge of the bounding box. Therefore, we can abstract the horizontal bounding box and utilize the inner tangent circle of the bounding box to represent the different ground pixel weight distribution in the bounding box. Let the horizontal bounding box $R=\left(x_{c},y_{c},w,\;h\right)$ , where $x_{c}$ and $y_{c}$ represent the horizontal and vertical coordinates of the center of the bounding box, w and h represent the width and height of the bounding box, respectively. At this time $\left(\mu_{x},\mu_{y}\right)$ represents the center coordinates of the ellipse, and $\rho_{x}$ , $\rho_{y}$ are the lengths of the semiaxis of the ellipse along the x and y axes, respectively. Correspondingly, $\mu_{x}=x_{c}$ , $\mu_{y}=y_{c}$ , $\rho_{x}=\frac{w}{2}$ , $\rho_{y}=\frac{h}{2}$ , the corresponding ellipse equations are:


(13) $$\begin{array}{c} \frac{{\left(x-\mu_{x}\right)}^{2}}{\rho_{x}^{2}}+\frac{{\left(y-\mu_{y}\right)}^{2}}{\rho_{y}^{2}}=1\; \end{array}$$

The probability density function for a p-dimensional random vector $\chi ={\left(X_{1},\cdots ,X_{p}\right)}^{T}$ can be written as Equation 14:


(14) $$\begin{array}{c} f\left(\chi |\mu ,\Sigma \right)=\frac{exp\left(-\frac{1}{2}{\left(\chi -\mu \right)}^{\tau }\Sigma^{-1}\left(\chi -\mu \right)\right)}{\sqrt{{\left(2\pi \right)}^{p}}{\left|\Sigma \right|}^{\frac{1}{2}}} \end{array}$$

The distribution defined by this function is the p-element normal distribution,denoted as χ~N(μ,Σ) , where $\Sigma^{-1}$ denotes the inverse matrix of Σ , |Σ| denotes the determinant of Σ , and ${\left(\chi -\mu \right)}^{\tau }$ denotes the transpose of the vector (χ−μ) . Based on the Mahalanobis distance we get that when ${\left(\chi -\mu \right)}^{\tau }\Sigma^{-1}\left(\chi -\mu \right)=1$ , the Equation 13 is then the contour of a two-dimensional Gaussian distribution. At this point, the horizontal bounding box $R=\left(x_{c},y_{c},w,\;h\right)$ can be modeled as a two-dimensional Gaussian distribution N(μ,Σ) with $\text{μ}=\left[\begin{array}{c} x_{c}\\ y_{c} \end{array}\right]$ , $\text{Σ}=\left[\begin{array}{c} \begin{array}{cc} \frac{w^{2}}{4} & 0 \end{array}\\ \begin{array}{cc} 0 & \frac{h^{2}}{4} \end{array} \end{array}\right]$ and the similarity between the bounding boxes A $\left(x_{a},y_{a},w_{a},\;h_{a}\right)$ and B $\left(x_{b},y_{b},w_{b},\;h_{b}\right)$ can be converted into the distribution distance between two Gaussian distributions. For the two-dimensional Gaussian distributions $\mu_{a}=N\left({\text{m}}_{1},\Sigma_{1}\right)$ and $\mu_{b}=N\left({\text{m}}_{2},\Sigma_{2}\right)$ for both A and B, define the two-dimensional Wasserstein Distance between the two as Equations 15–18:


(15) $$\begin{array}{c} W_{2}^{2}\left(\mu_{a},\mu_{b}\right)={\left\|m_{1}-m_{2}\right\|}_{2}^{2}+Tr\left(\Sigma_{1}+\Sigma_{2}-2{\left(\Sigma_{2}^{\frac{1}{2}}\Sigma_{1}\Sigma_{2}^{\frac{1}{2}}\right)}^{\frac{1}{2}}\right)\; \end{array}$$

(16) $$\begin{array}{c} ={\left\|m_{1}-m_{2}\right\|}_{2}^{2}+\frac{{\left(w_{1}-w_{2}\right)}^{2}+{\left(h_{1}-h_{2}\right)}^{2}}{4}\; \end{array}$$

(17) $$\begin{array}{c} ={\left\|m_{1}-m_{2}\right\|}_{2}^{2}+{\left\|\Sigma_{1}^{\frac{1}{2}}-\Sigma_{2}^{\frac{1}{2}}\right\|}_{F}^{2} \end{array}$$

(18) $$\begin{array}{c} ={\left\|\left({\left[cx_{a},cy_{a},\frac{w_{a}}{2},\;\frac{h_{a}}{2}\right]}^{T},{\left[cx_{b},cy_{b},\frac{w_{b}}{2},\;\frac{h_{b}}{2}\right]}^{T}\right)\right\|}_{2}^{2} \end{array}$$

Where ${\left\|\cdot \right\|}_{F}$ is the F-parameter of the matrix. Finally, $W_{2}^{2}\left(\mu_{a},\mu_{b}\right)$ is normalized to obtain the final metric, which is collated to obtain the final EWDIoU formula as Equation 12. where M is a constant with respect to the dataset, in our experiments, we compared the effect of different values of M on the results, and finally achieved the best results with M=1.0 .

#### 2.3.4 Evaluation metrics

This section outlines the evaluation metrics employed to comprehensively assess the performance of the small tomato detection model. The primary metrics include precision (P), recall (R), mean average precision (mAP), floating point operations per second (FLOPs), number of network parameters, and inference speed.


(19) $$\begin{array}{c} P=\frac{TP}{TP+FP} \end{array}$$

(20) $$\begin{array}{c} R=\frac{TP}{TP+FN} \end{array}$$

(21) $$\begin{array}{c} AP=\int_{0}^{1}P\left(R\right)dR \end{array}$$

(22) $$\begin{array}{c} mAP=\frac{\int_{q=1}^{Q}AP\left(q\right)}{Q} \end{array}$$

In Equations 19, 20, the calculation of precision(P) and recall(R) relies on three key metrics: true positives (TP), false positives (FP), and false negatives (FN). When the model successfully identifies a small tomato target, it is recorded as TP, whereas FP and FN represent, respectively, the number of false detections of nonexistent targets and the number of missed detections of actual targets by the model. Precision (P) measures the model’s capability to correctly identify small tomato targets among all predicted targets, while recall (R) assesses the proportion of actual targets successfully detected by the model. For the detection performance of small tomatoes in each category, a precision-recall (P-R) curve can be plotted, with the average precision (AP) defined as the area under the curve. The closer the AP value is to 1, the better the model’s detection performance for that specific category. The mean average precision (mAP), calculated as the weighted average of the AP values across all categories, is a widely adopted performance evaluation metric in target detection tasks. It provides a visual and comprehensive representation of the model’s overall performance, where Q in the equation represents the total number of target categories. Moreover, model complexity is typically quantified by the number of floating-point operations (FLOPs), which represents the computational resources required by the model and serves as a crucial metric for assessing algorithmic efficiency. The speed of target detection is measured in frames per second (FPS), with a higher FPS value indicating superior real-time processing capability. A comprehensive evaluation of these metrics offers a thorough and rigorous assessment of the model’s performance, enabling multidimensional comparison and optimization.

#### 2.3.5 Environmental settings

The proposed model was trained and tested using the small tomato dataset in field scenarios with a total of 7332 images. The specific training environment is Intel(R) Xeon(R) Gold 6248R@3.00GHz processor with an NVIDIA GeForce RTX4090 graphics card. The deep learning modeling framework uses Pytorch 2.4.1 and Python 3.8.19, the CUDA version was selected as 11.7, and the operating system was selected as Windows 11. All experiments were trained for 300 epochs with the following hyperparameters: a Adaptive Moment Estimation (Adam) optimizer with a batch size of 4, an initial learning rate of 0.001, a momentum factor of 0.937, and a weight decay of 0.0005. In order to ensure the fairness and comparability of the model effects, we tried to use the same parameter settings for both the comparison and ablation experiments, and some important hyper-parameter settings are shown in **Table 2**.

**Table 2:** Training parameters settings.

Parameter	Value	Parameter	Value
Epoch	300	Initial Learning Rate	1 × 10^−3^
Batch size	4	Weight-Decay	5 × 10^−4^
Optimizer	Adam	Momentum	0.937

## 3 Results

### 3.1 Improving test results via data enhancement

To expand the training samples and enhance the model’s generalization ability, robustness, and adaptability in real-world applications, we apply data augmentation techniques such as exposure adjustment, rotation, blurring, random brightness variation, and mirroring to simulate diverse scene variations. The experimental results are presented in **Table 3**.

**Table 3:** Comparison of results before and after data augmentation.

Model	Class	P(%)	R(%)	mAP50(%)
YOLOv8n	all	87.0	75.6	82.4
	gtomato	85.4	68.7	78.1
	rtomato	85.4	78.1	82.2
	ytomato	90.2	80.8	87.1
YOLOv8n(without enhancement)	all	79.9	76.2	81.6
	gtomato	83.4	74.7	82.0
	rtomato	78.5	84.9	88.0
	ytomato	77.7	69.0	74.0

After data augmentation, the overall mAP@50 showed a noticeable improvement, indicating enhanced detection performance of the model. In addition, the precision values for all categories and the overall precision increased, suggesting a reduction in false positives and more effective feature learning. While the recall of the ytomato class improved, the recall of other categories slightly declined. This is likely due to increased background complexity in the augmented images, which made the model more conservative in its predictions, leading to a higher miss rate for true targets. However, since the ytomato class had relatively few samples before augmentation, the augmented data effectively alleviated the issue of data scarcity and helped the model learn more stable features. Although the mAP of gtomato and rtomato slightly decreased—possibly due to distributional shifts or reduced feature stability caused by augmentation—the overall improvement in mAP indicates that the model became more balanced and achieved better generalization.

### 3.2 Ablation study

In this section, the fully enhanced model is compared with simplified variants incorporating individual improvements to independently evaluate the effectiveness of each component. All enhancements are built upon the baseline YOLOv8n model, targeting the following aspects: modifications to the YOLOv8n backbone; introduction of a novel feature pyramid pooling structure, DASPPF; incorporation of a lightweight detection head; integration of the CSAM attention mechanism to improve multi-scale feature fusion; and replacement of the traditional loss function with the proposed EWDIoU loss, which leverages a two-dimensional Gaussian distribution to enhance bounding box regression. In **Table 4**, each improvement was incrementally incorporated into the baseline model, and the corresponding performance metrics were evaluated. Specifically, “A” denotes the backbone enhancement, “B” refers to the proposed DASPPF module, “C” indicates the addition of a small object detection head, and “D” represents the proposed CSAM module. The final model, Ta-YOLO, integrates all these enhancements.

**Table 4:** Results of ablation experiments.

Model	Class	P(%)	R(%)	mAP50(%)	mAP50-90(%)	Params(M)	FLOPs(G)	FPS (frames/s)
YOLOv8n	all	87.0	75.6	82.1	45.6	11.48	8.1	120.11
	gtomato	85.4	68.7	78.1	41.1			
	rtomato	85.4	78.1	82.2	48.5			
	ytomato	90.2	80.8	87.1	47.2			
YOLOv8+A	all	87.7	73.1	81.2	43.9	8.5	10.2	200.0
	gtomato	85.7	65.2	76.8	40.1			
	rtomato	88.7	77.4	85.7	49.8			
	ytomato	88.6	76.7	81.1	41.7			
YOLOv8+A+B	all	85.1	75.1	82.4	44.5	9.96	10.2	168.2
	gtomato	83.4	70.1	78.6	40.9			
	rtomato	86.5	82.4	88.0	50.9			
	ytomato	85.5	72.7	80.5	41.6			
YOLOv8+A+B+C	all	85.9	75.4	83.3	45.5	9.62	14.2	156.3
	gtomato	84.6	70.6	80.1	42.4			
	rtomato	85.4	78.9	86.4	50.7			
	ytomato	87.4	76.7	83.4	43.8			
YOLOv8+A+B+C+D	all	85.9	76.0	84.0	47.0	10.57	14.3	153.9
	gtomato	84.6	71.6	80.9	43.1			
	rtomato	88.4	79.5	87.9	52.4			
	ytomato	84.4	77.0	83.2	45.5			
Ta-YOLO	all	86.7	76.9	84.4	45.9	10.58	14.3	131.58
	gtomato	86.0	70.8	81.0	43.2			
	rtomato	87.5	79.0	87.2	51.5			
	ytomato	86.7	75.5	84.9	43.1			

**Figure 9:**
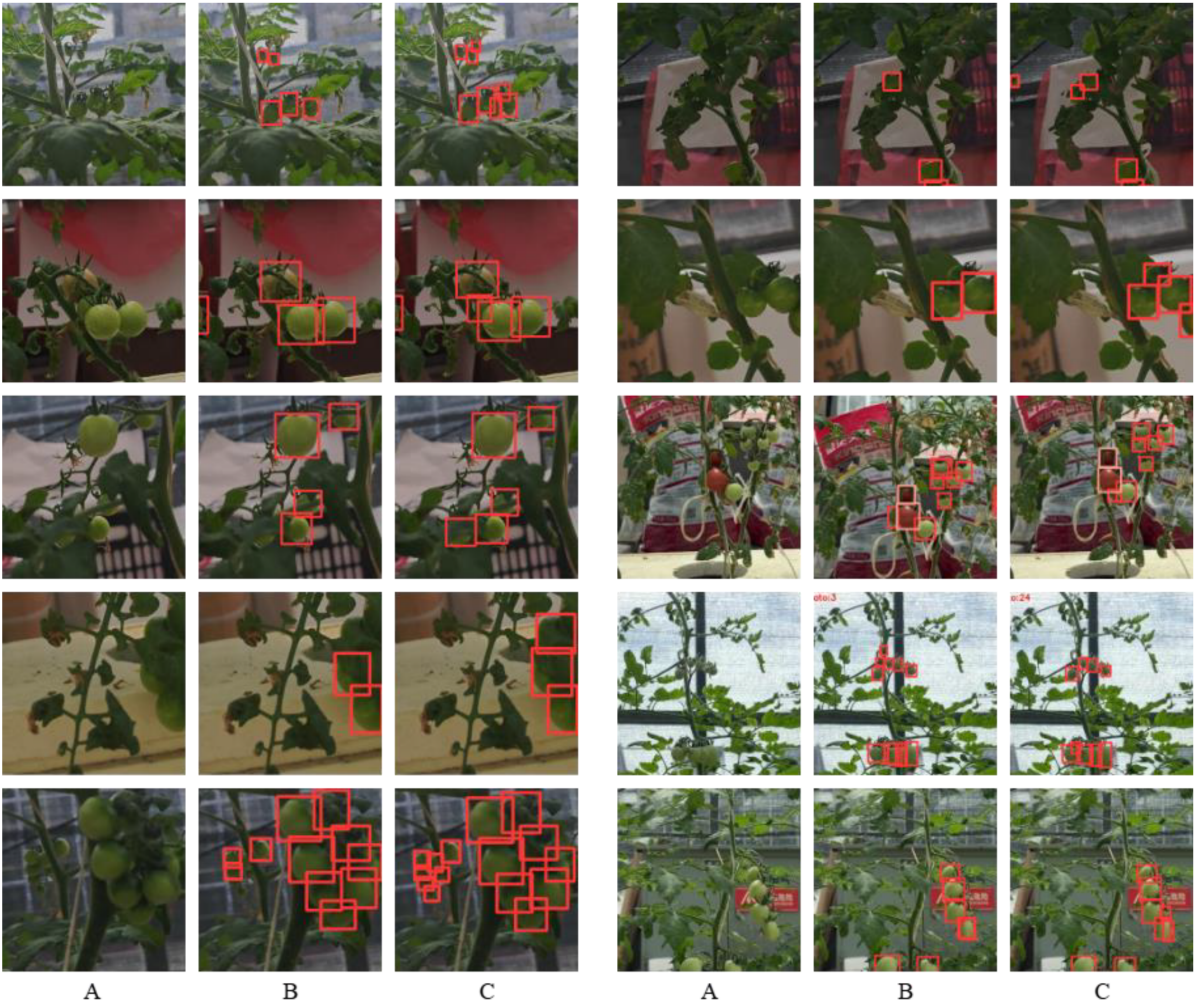
Example of detection results under different occlusion scenarios. **(A)**Original images, **(B)** benchmark model, **(C)** Ta-YOLO.

The results of the comprehensive ablation study are summarized in the table, highlighting the following key findings: (1) Lightweight modifications to the backbone successfully reduced the parameter count and increased inference speed (FPS), albeit at the cost of reduced accuracy. (2) The proposed DASPPF feature pyramid pooling structure significantly enhanced the extraction of salient features, with recall rates for green and red tomatoes reaching 70.1% and 82.4%, respectively—improvements of 1.4% and 4.3% over the baseline. Furthermore, the mAP increased by 0.5% and 5.8% compared to the baseline. These results indicate that preserving global contextual information in complex real-world scenes facilitates more accurate target recognition. (3) The addition of the tiny detection head increased the mAP to 83.3%, while simultaneously reducing the parameter count and improving FPS relative to the baseline. However, this enhancement resulted in an increased computational load. These findings indicate an improved multi-scale detection capability, rendering the model more effective for small tomato detection. (4) The CSAM attention mechanism further enhanced recognition accuracy by efficiently integrating multidimensional feature information, particularly benefiting the detection of multiple small tomatoes at image edges or under occlusion. Moreover, the proposed EWDIoU loss function effectively addressed challenges associated with small target detection, yielding superior performance across small tomato categories. Across all evaluated samples, the mAP for heavily shaded green and red tomatoes improved from 78.1% and 82.2% to 81.0% and 87.2%, respectively, demonstrating the targeted effectiveness of our approach in mitigating shading-related challenges. Furthermore, a comparative analysis between the original baseline and Ta-YOLO under complex real-world conditions, including occlusion, is presented in **Figure 9**. The results confirm that Ta-YOLO achieves superior detection performance in these challenging scenarios.

### 3.3 Comparative experiments

The two-stage detection algorithm initially extracts candidate regions from the input image, followed by classification of each candidate. To evaluate the effectiveness of the proposed method, it was compared against the classical two-stage detector Faster R-CNN. Additionally, the enhanced model was benchmarked against several widely used one-stage detection algorithms, including YOLOv5, YOLOv7, YOLOv9, YOLOv11, and HyperYOLO. To ensure fairness and emphasize the effectiveness of the proposed method, comparisons were performed using lightweight variants of the evaluated algorithms. The experimental results, presented in **Table 5**, indicate that Ta-YOLO achieved recall and mAP values of 76.9% and 84.4%, respectively, outperforming most competing models. Significantly, YOLOv9s attained the highest mAP of 85.3% in this comparison. However, YOLOv9s exhibited a parameter count of 37.88 million and a computational complexity of 40.6 GFLOPs, exceeding those of Ta-YOLO by 27.3 million parameters and 26.1 GFLOPs, respectively. Additionally, YOLOv9s demonstrated lower FPS performance compared to Ta-YOLO, underscoring the trade-off between accuracy and computational efficiency. Although YOLOv7’s mAP was only 0.4% lower than that of Ta-YOLO, its parameter count was approximately thirteen times greater. Compared to YOLOv11 and HyperYOLO, Ta-YOLO achieves higher FPS with a similar parameter count, while demonstrating superior accuracy.

**Table 5:** Comparison results with different target detectors.

Model	Class	P(%)	R(%)	mAP50(%)	Params(M)	FLOPs(G)	FPS(frames/s)
Faster R-CNN	all	38.0	50.4	46.89	136.73	369.7	18.5
	gtomato	35.63	61.71	45.96			
	rtomato	41.91	67.21	56.23			
	ytomato	36.73	54.27	38.47			
YOLOv5	all	91.0	56.6	66.29	26.81	16.0	34.3
	gtomato	91.07	55.60	67.0			
	rtomato	85.96	63.30	68.9			
	ytomato	98.18	50.97	62.96			
YOLOv7	all	88.0	77.6	84.0	141.93	105.1	111.1
	gtomato	86.0	75.3	82.2			
	rtomato	87.4	77.4	81.7			
	ytomato	93.6	79.7	88.1			
YOLOv8n	all	87.0	75.6	82.4	11.48	8.1	120.11
	gtomato	85.4	68.7	78.1			
	rtomato	85.4	78.1	82.2			
	ytomato	90.2	80.8	87.1			
YOLOv9s	all	89.5	79.2	85.1	37.88	40.6	110.7
	gtomato	85.3	75.9	82.7			
	rtomato	87.8	80.7	85.0			
	ytomato	95.3	81.0	90.2			
YOLOv11n	all	86.4	73.7	81.4	9.85	6.3	303.3
	gtomato	83.6	69.8	78.0			
	rtomato	84.4	76.4	81.1			
	ytomato	91.3	74.9	85.0			
	all	86.2	75.2	82.2	10.38	7.6	204.8
HyperYOLO	gtomato	83.2	70.4	78.1			
	rtomato	83.6	78.4	81.6			
	ytomato	91.1	77.0	86.9			
Ta-YOLO	all	86.7	76.9	84.4	10.58	14.3	131.58
	gtomato	86.0	70.8	81.0			
	rtomato	87.5	79.0	87.2			
	ytomato	86.7	75.5	84.9			

Nevertheless, examination of the table reveals that, despite Ta-YOLO’s superior overall performance compared to other detectors, its recall for green tomatoes is below the average recall, indicating the presence of false negatives in green tomato detection. Moreover, this issue is not unique to Ta-YOLO but is prevalent across most detection models. An analysis of the dataset revealed that extensive leaf shading on green tomatoes contributes to erroneous detections. Notably, the dataset was annotated with stringent criteria, including labeling tomatoes even when heavily occluded by foliage, which may further contribute to the detection challenges observed. It is worth noting that, YOLOv9 achieves a relatively higher recall for green tomatoes. Our analysis attributes this to YOLOv9’s heavier parameterization, which facilitates more precise alignment of feature map edges. Consequently, future work will focus on enhancing edge and texture perception by improving the extraction and representation of edge features.

**Figure 10:**
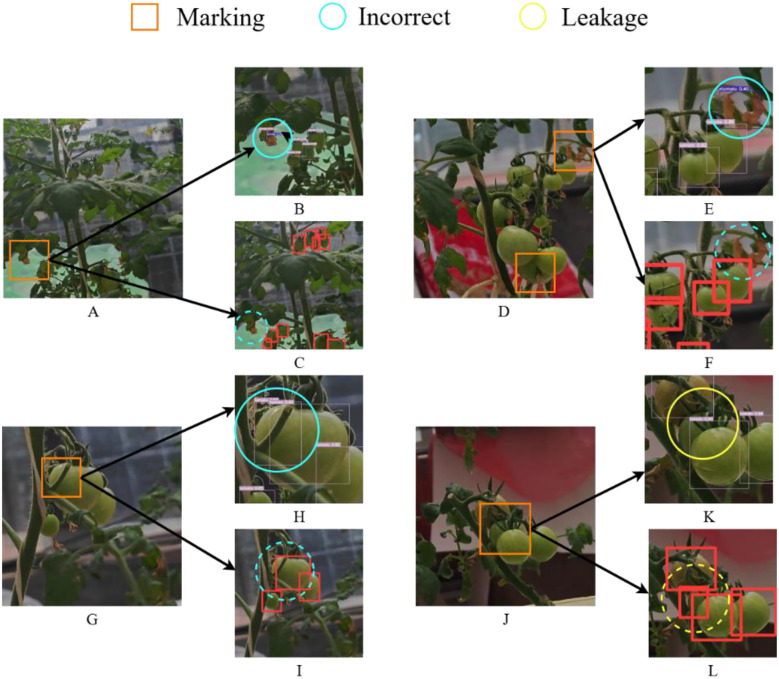
Visual comparison figure of YOLOv7 and Ta-YOLO detection results in four extreme environments. **(A, D, G, J)** showed four different detection situations. **(B, E, H, K)** displayed the YOLOv7 detection results. **(C, F, I, L)** presented the Ta-YOLO detection results.

**Figure 11:**
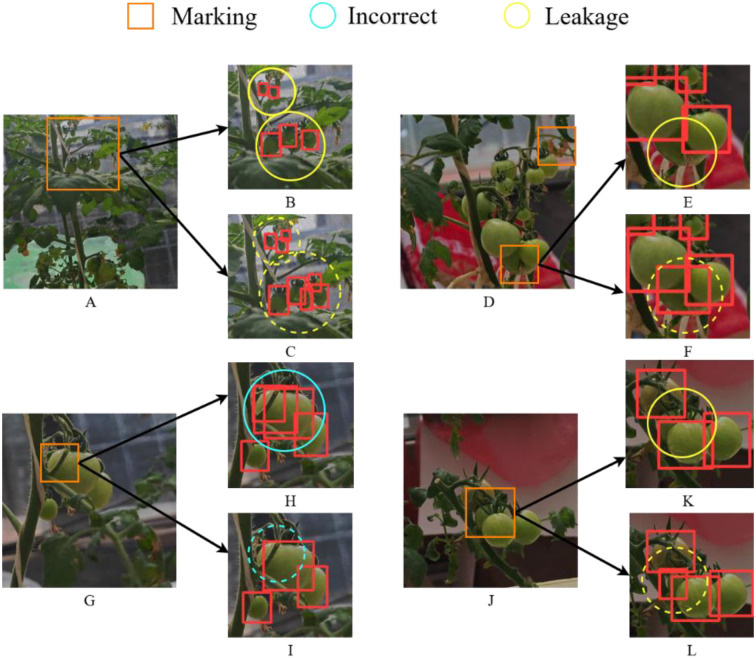
Visual comparison figure of YOLOv8 and Ta-YOLO detection results in four extreme environments. **(B, E, H, K)** displayed the YOLOv8 detection results. **(C, F, I, L)** presented the Ta-YOLO detection results.

In this section, four representative challenging cases from the dataset were selected, as illustrated in **Figures 10A, D, G, J**. In these figures, yellow circles denote missed detections, blue circles indicate false positives, and orange squares mark regions with increased identification difficulty. In **Figure 10B, E**, the leaves highlighted by blue circles were erroneously classified as green and red tomatoes, respectively. In comparison, the proposed algorithm correctly avoids these misclassifications, as demonstrated by the absence of false positives within the blue dashed circles in **Figure 10C, F**. In **Figure 10H**, occlusion caused by tomato branches adversely affects detection, resulting in the tomato marked by the blue circle being erroneously identified as multiple instances. In contrast, the corresponding region within the blue dashed circle in **Figure 10I** is correctly detected by the proposed method. Likewise, in **Figure 10J**, extensive occlusion from the tomato petiole leads to a missed detection of the small tomato indicated by the yellow circle in **Figure 10K**, whereas **Figure 10L** shows successful recognition. The same four challenging cases presented in **Figure 10** are used to visualize and compare the detection results between YOLOv8 and Ta-YOLO in **Figures 11A, D, G, J**. In **Figure 11B**, the very small tomatoes indicated by yellow circles were completely missed, whereas those within the yellow dashed circles in **Figure 11C** were accurately detected, including occluded instances. In **Figure 11E**, the tomato enclosed by the yellow circle was heavily obscured and not correctly identified; however, the improved algorithm presented in this study successfully detected it in **Figure 11F**. Similarly, **Figure 11H** exhibits the same issue observed in **Figure 10H**, where the tomato marked by the blue circle was mistakenly identified as multiple instances, whereas the corresponding region in **Figure 11I** within the blue dashed circle was correctly recognized. In **Figure 11J**, occlusion caused by the tomato petiole led to a missed detection of the small tomato marked by the yellow circle in **Figure 11K**, while **Figure 11L** demonstrates its accurate detection. Collectively, these results demonstrate that Ta-YOLO achieves higher accuracy and greater robustness in detecting shaded small tomatoes under real production conditions.

### 3.4 Comparative experiments on different attentional

**Figure 12:**
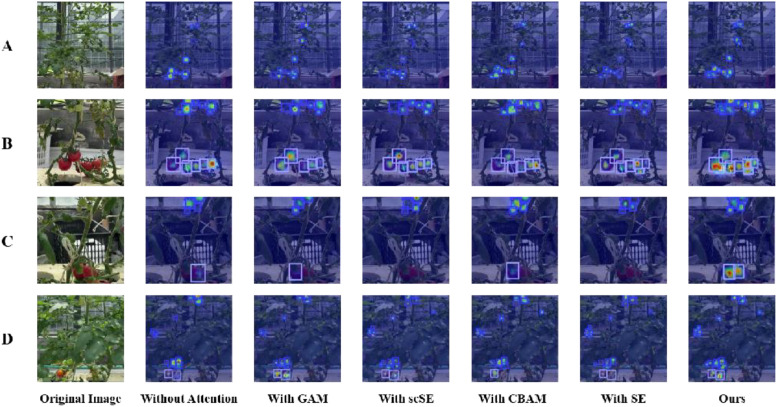
Heatmap visualizations of small tomato detection under different attention mechanisms. **(A)** Example of Extremely Small Tomato; **(B)** Example of Occlusion by Stem; **(C)** Example of Occlusion by Leaf; **(D)** Example of Inter-Class Occlusion.

In deep learning, the attention mechanism, which simulates the selective focus of human cognition, has been extensively applied across domains including image processing and natural language processing. Among various attention mechanisms, the Global Attention Module (GAM) stands out as a global attentional approach that effectively preserves the majority of salient information, thereby enhancing feature interaction. The SE attention mechanism emphasizes effective feature extraction by employing a squeeze-and-excitation process that encourages the network to integrate spatial and channel information within the local receptive field. Differently, the scSE attention mechanism simultaneously combines spatial and channel attention in parallel to enhance feature representation. Similarly, the CBAM attention mechanism facilitates feature interaction through sequential fusion of channel and spatial attention. Each of these attention mechanisms, having demonstrated strong performance across various tasks, was integrated into the Ta-YOLO model for comparative evaluation against CSAM. The results showed that, CSAM achieved the highest accuracy, with mAP and recall values of 84.4% and 76.9%, respectively. Among the competing mechanisms, CBAM exhibited the lowest parameter count and computational cost, with 9.62 million parameters and 14.2 GFLOPs, although its mAP and recall were 1% and 2% lower than those of CSAM. GAM, despite having the largest parameter count at 15.88 million, attained an mAP of 83.4%, comparable to CBAM, thus neither surpassing CSAM’s performance nor justifying the increased complexity. Additionally, CSAM maintains the same plug-and-play compatibility as these established attention modules. **Table 6** presents the detection performance of Ta-YOLO on the real tomato dataset, while **Figure 12** illustrates heatmap visualizations corresponding to different attention mechanisms. The results clearly indicated that the proposed CSAM module outperforms others by effectively concentrating on heavily occluded and small-sized tomatoes. In summary, the integration of spatial and channel attention within the CSAM module yields superior detection efficacy.

**Table 6:** Comparison of 5 different attention mechanism with metrics of mAP50, mAP50-90, precision, recall, Parameters and FLOPs.

Models	P (%)	R (%)	mAP50 (%)	mAP50-90 (%)	Params (M)	FLOPs (G)
Ta-YOLO_GAM	86.7	75.0	83.4	45.9	15.88	15.5
Ta-YOLO_SE	86.6	75.1	83.6	45.8	9.63	14.2
Ta-YOLO_scSE	86.8	74.4	82.7	45.1	9.89	14.4
Ta-YOLO_CBAM	86.3	74.9	83.4	45.7	9.62	14.2
Ta-YOLO_CSAM	86.7	76.9	84.4	45.9	10.58	14.3

### 3.5 Comparative experiments on different loss functions

The loss function plays a critical role in object detection tasks by quantifying the discrepancy between model predictions and ground truth labels. This measurement guides model optimization during training, ultimately improving detection performance. In this study, several widely used loss functions were evaluated on the Ta-YOLO model and compared against the proposed EWDIoU loss function to assess its effectiveness. The corresponding experimental results were summarized in **Table 7**. Among the evaluated loss functions, the EWDIoU achieved the highest mAP@50 and recall scores of 84.4% and 76.9%, respectively. Although the GIoU loss attained an identical recall value, its accuracy was lower at 83.0%, representing a 1.4% deficit compared to EWDIoU. The EIoU loss reached a mAP@50 of 83.8%, close to the highest value; however, its precision and recall metrics were inferior to those of the EWDIoU loss function. Although the original CIoU loss used in YOLOv8 achieved the highest precision of 88.2%, its mAP was limited to 82.3%, and recall reached only 75.1%. These results indicated that the model struggled to detect all valid targets, particularly small tomatoes with occluded edges, leading to missed detections. Consequently, this shortcoming contributed to the decline in both recall and mAP. The EWDIoU loss function employs a two-dimensional Gaussian distribution approach to process discretized data, effectively addressing the bounding box insensitivity to small tomato targets and thereby enhancing detection accuracy. The proposed improvements demonstrate notable performance gains, indicating that targeted enhancements can overcome common detection challenges, including performance degradation in complex environments characterized by occlusion and small targets, as well as reducing instances of misdetection and omission.

**Table 7:** Comparison of 5 different loss functions with metrics of mAP50 (Equation 16), precision (Equation 17), recall (Equation 19) and mAP50-90.

Models	P(%)	R(%)	mAP50(%)	mAP50-90(%)
Ta-YOLO-CIoU	88.2	75.1	82.3	45.7
Ta-YOLO-DIoU	87.4	76.7	82.9	45.2
Ta-YOLO-GIoU	86.6	76.9	83.0	45.7
Ta-YOLO-EIoU	86.4	76.0	83.8	45.6
Ta-YOLO-SIoU	85.7	73.2	83.2	46.1
Ta-YOLO-EWDIoU	86.7	76.9	84.4	45.9

### 3.6 Experiments with different values of λ in the EWDIoU function

**Figure 13:**
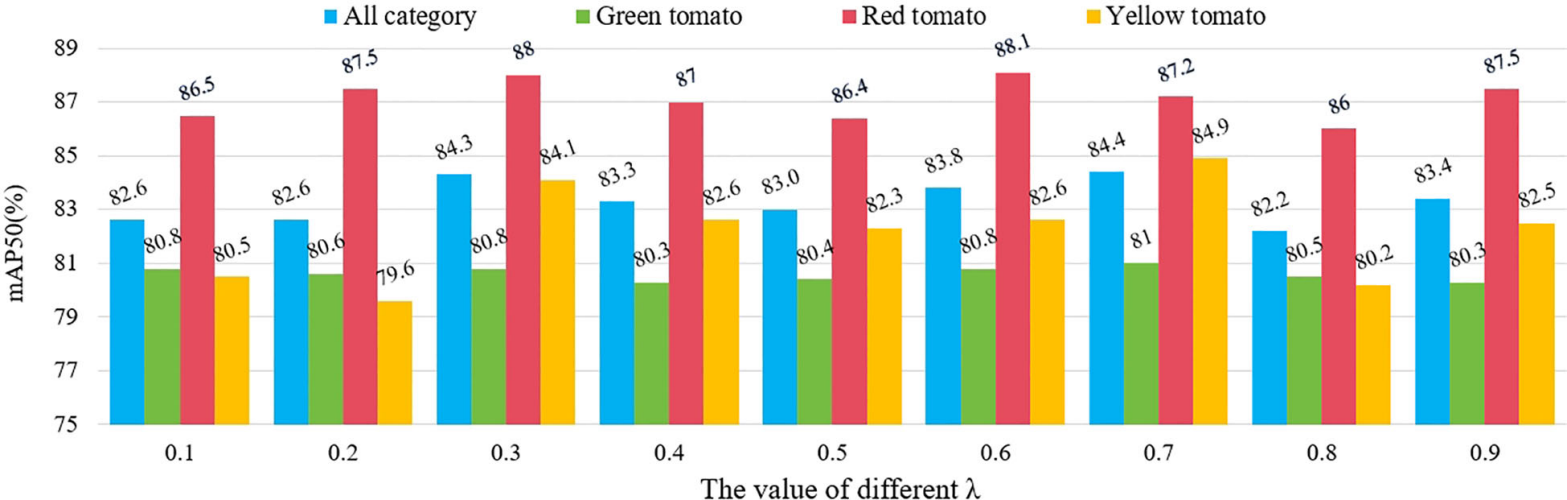
mAP values of different classes of small tomatoes at different λ -values.

In the proposed EWDIoU loss function, to effectively mitigate the impact of small target tomato bounding boxes on the loss calculation while preserving the detection performance advantages for larger target tomatoes, an adjustable hyperparameter λ was introduced. This hyperparameter balances the contribution of the IoU in the loss function calculation, allowing for adaptive adjustments across different target scales. On the one hand, λ suppresses the bias amplification effect caused by the smaller scale of small targets in bounding box errors. On the other hand, it ensures the importance of large targets is preserved in the detection task, thereby achieving a dynamic balance and optimizing the loss function’s performance for detecting targets of varying scales. The model’s robustness and accuracy in handling multi-scale targets were significantly enhanced. We conducted experiments with 10% intervals and keeping the criterion of $\lambda_{1}+\lambda_{2}=1$ to observe the effects brought by different values on the overall detection results, as shown in **Figure 13**. The number of experiments is 9 groups in total, and the experimental results showed that when $\lambda_{1}$ is 0.7 and $\lambda_{2}$ is 0.3, our EWDIoU effect performed the best on the small tomato dataset of the field scene, and its total category mAP value reached 84.4%. In the comparison across different categories, the mAP trends of green-fruited tomatoes and yellow-fruited tomatoes exhibited high consistency with the overall category mAP. With the highest mAP in the total category, green-fruited tomatoes and yellow-fruited tomatoes reached 81% vs. 84.9% mAP, respectively. Our data analysis showed that green-fruited tomatoes had a higher probability of being obscured in the sample of obscured small tomatoes, and that the number of green-fruited tomatoes in the sample of small targets was relatively large. Therefore, when the mAP of green-fruited tomato reached the optimum, the mAP of the total category also reached the peak, which further validated that the EWDIoU loss function is able to effectively solve the problem of the detection of occluded fruits. In addition, in the practical application scenario, the shading rate of yellow-fruited tomato was much lower than that of green-fruited tomato, but its mAP was still able to keep the same trend with the total category mAP, which indicated that the loss function has strong robustness and adaptability. The change in mAP of red-fruited tomato was relatively smooth, and the fluctuation range of its mAP was controlled within 2.1%. This phenomenon can be primarily attributed to the relatively moderate shading in red-fruited tomato samples, coupled with greater color variability arising from differing ripeness levels. Nonetheless, the consistent detection performance suggests that the EWDIoU loss function does not induce significant errors or overfitting when applied to this category. In summary, EWDIoU demonstrates strong adaptability and stability across varying degrees of occlusion scenarios.

## 4 Discussion

Accurate counting of small tomato fruits in real-world production environments poses significant challenges and is of substantial practical importance. It is imperative to reduce the labor costs associated with manual counting while mitigating errors arising from the diminutive size of tomato targets and inter-fruit shading. This study proposed a detection and counting method specifically tailored for densely planted small tomatoes under realistic cultivation conditions. The algorithm maintains the integrity of small tomato targets within unaltered field images—without necessitating image zooming or cropping—thereby enabling effective and reliable detection.

The algorithm used yolov8n as the baseline model, and used the C2f_Repghost module and the SPDC module to adjust the structure of the backbone network in the original algorithm, so that the model could reduce the amount of computation and maintain the lightweight while enhancing the feature information extraction ability for small targets, so as to cope with the occlusion problem more effectively. And the new DASPPF structure was proposed to use average pooling to reduce the influence of redundant information on effective features and further improve the quality of feature extraction in the case of occlusion. Meanwhile, the CSAM multiple attention structure was constructed to introduce spatial and channel attention mechanisms after decomposing the input information to realize the deep fusion of feature maps at different scales. In addition, a new formulation of the EWDIoU loss function was proposed that utilized a two-dimensional Gaussian distribution function to abstract the original IoU loss function, which solved the problem of insensitivity of the original IoU to small target detection and effectively improves the performance of small tomato detection in the case of occlusion. Finally, an additional small detection head was incorporated into the detector architecture to enhance the extraction of fine-grained features, thereby improving the recognition of small targets. Experimental results demonstrate that the proposed Ta-YOLO model achieves high accuracy and robustness in addressing the occlusion challenges inherent in small tomato counting within real production environments. Compared to the original baseline, Ta-YOLO exhibits significant improvements in both accuracy and recall, alongside enhanced global feature extraction and superior small target detection performance.

And why did we choose YOLO as the baseline model and not use other lightweight models? As a single-stage detector, YOLO is well-suited for real-time video analysis in agricultural environments, where rapid and continuous detection is required. In contrast, models such as MobileNet-SSD offer faster inference but tend to underperform in complex scenes, while Transformer-based detectors like DETR are resource-intensive and less suitable for real-time deployment. Moreover, YOLO benefits from extensive open-source support and compatibility with deployment toolchains (e.g., TensorRT, ONNX), which significantly simplifies engineering implementation. Designing a lightweight model from scratch would introduce challenges such as lack of pre-trained weights and increased risk of overfitting, particularly in data-limited agricultural scenarios. For these reasons, we chose to adopt and tailor YOLO through lightweight modifications, balancing performance, efficiency, and practical.

Despite its advantages, Ta-YOLO has certain limitations. As shown in **Table 2**, while Ta-YOLO attains a high recognition accuracy of 87.2% for red-fruited tomatoes, its accuracy for yellow-fruited tomatoes is 5.3% lower than that of YOLOv9s. This notable gap contributes to an overall detection accuracy that is lower than YOLOv9s. A likely cause for this discrepancy is data imbalance; constraints in the actual production environment and the short growth cycle of small tomatoes resulted in fewer images containing yellow-fruited tomatoes during data collection. Consequently, the dataset contained fewer samples of yellow-fruited tomatoes compared to green- and red-fruited varieties. What’s more, inaccuracies in manual annotation during dataset preparation may have led to misclassifications, especially for small tomatoes exhibiting intermediate colors during their developmental stages. Therefore, expanding the dataset and refining the maturity category definitions would be beneficial. Secondly, to preserve the natural growth state of small tomatoes and effectively address the influence of leaf shading on counting in actual production, we deliberately avoided regional cropping or other image preprocessing techniques. Instead, the full appearance of small tomatoes as seen in the production environment was retained. Although this approach increased detection difficulty, it enhanced Ta-YOLO’s applicability and robustness in real-world agricultural scenarios.

Overall, Ta-YOLO represents a significant advancement for real-world production settings, particularly in the detection and counting of small tomatoes under occlusion. Its demonstrated accuracy, efficiency, and robustness provide a practical solution for improving the commercial productivity of agricultural operations. Moreover, Ta-YOLO effectively balances detection speed and precision, underscoring its potential to supplant labor-intensive manual counting. Future work will aim to further optimize the model and investigate its scalability across other small-target crop species and diverse application contexts.

## Data Availability

The raw data supporting the conclusions of this article will be made available by the authors, without undue reservation.
